# Disruptions of cytological screening procedures due to the COVID-19 pandemic in Slovakia: Juliana Melichová

**DOI:** 10.1093/eurpub/ckac129.255

**Published:** 2022-10-25

**Authors:** J Melichova, E Marusakova, L Bilikova, P Sivčo, D Plancikova, D Hazerova, T Pham, S Paulik, S Putekova, M Majdan

**Affiliations:** Department of Public Health, Trnava University in Trnava, Trnava, Slovakia

## Abstract

**Background:**

During the COVID-19 pandemic, most settings experienced healthcare service disruptions. The majority of cytological screening procedures were postponed to focus on assisting patients infected with COVID-19. In this study, we aimed to analyse the impact of the impact of the COVID-19 pandemic on the uptake of cervical cancer screening in Slovakia.

**Methods:**

Data on cytological screening procedures were obtained from two of the three health insurance companies in Slovakia for the years 2019 and 2020, covering the population of women aged 15 and older. All data were calculated stratified for age groups. Rates of cytological screenings were calculated as the number of procedures per women registered in the insurance company in the same age group and rate ratios were calculated as ratios of the rates for the years 2020 and 2019 for the same age group. Incidence rates were calculated as the number of newly diagnosed cervical cancer cases per women registered in the insurance company in the respective year.

**Results:**

Rate ratios of cytological screening procedures revealed that in both examined health insurance companies, the rate of cytological exams was lower in 2020 compared to 2019 (0.95 and 0.89). This was observed across all age groups. The results showed a clear and statistically significant age gradient, indicating that the level of disruption increased with age. The age group 60-69 years had the highest incidence rate of cervical cancer in 2019, at 54.3 per 100 000. In 2020, the highest rate was 48.3 in the age group 50-59 years. The lowest rates were in children and young adults (<20 years).

**Conclusions:**

This study confirms the significant impact of the pandemic on cervical cancer screening uptake in Slovakia, which may have delayed the diagnosis of cervical cancer into later stages of the disease with a worse prognosis. This may lead to increased mortality and years lived with disability due to this disease in Slovakia.

**Key messages:**

• Disruptions in cervical cancer screenings were observed in Slovakia during the COVID-19 pandemic which may result in an increase in cervical cancer incidence and mortality.

• Strategies should be implemented to maintain cancer screening programs during health emergencies to avoid excessive mortality and morbidity.

